# Self-Distancing Reduces Probability-Weighting Biases

**DOI:** 10.3389/fpsyg.2018.00611

**Published:** 2018-06-13

**Authors:** Qingzhou Sun, Huanren Zhang, Liyang Sai, Fengpei Hu

**Affiliations:** ^1^Institute of Brain and Management Sciences, College of Economics and Management, Zhejiang University of Technology, Hangzhou, Zhejiang, China; ^2^Social Science Division, New York University Abu Dhabi, Abu Dhabi, United Arab Emirates; ^3^Institute of Psychological Science, Zhejiang Key Laboratory for Research in Assessment of Cognitive Impairments, Center for Cognition and Brain Disorders, Hangzhou Normal University, Hangzhou, China

**Keywords:** self-distancing, self-immersing, valuation task, probability-weighting bias, probability-weighting function

## Abstract

We have abundant evidence that people exhibit biases in weighting probability information. The current study aims to examine whether self-distancing would reduce these biases. Participants in this study were instructed to use either a self-distancing or a self-immersing strategy to regulate their reasoning when they indicated their valuations of different lotteries. The results show that, compared to the baseline group, participants in the self-distancing group exhibited less distortion in the probability-weighting function, while those in the self-immersing group exhibited more distortion. These results offer evidence for the power of self-distancing in reducing probability-weighting biases.

## Introduction

People often exhibit biases in weighting probabilistic events—that is, overweighting small probabilities and underweighting large probabilities (for a review, see Fox and Poldrack, 2009). Sometimes, these biases lead to irrational and maladaptive behaviors, such as irrational investment in the market (Kliger and Levy, 2008), inaccurate diagnoses of medical conditions (Collins and Huynh, 2014), and unreasonable government budgets (McGraw et al., 2011). Given the adverse effects of these behaviors on individuals and society, it is important to understand how to reduce these biases.

Studies have identified several factors that can influence probability-weighting biases, including presentation formats (Visschers et al., 2009), construal levels (Trautmann and van de Kuilen, 2012; Lermer et al., 2016), and real vs. hypothetical payment (Deckop et al., 2004; Smith et al., 2009). In this study, we investigate whether self-distancing can reduce the probability-weighting biases.

Self-distancing refers to individuals' cognitive strategy of assuming a third-person perspective to psychologically remove themselves from the events that happen to them (Kross et al., 2005; Kross and Ayduk, 2017). When people deal with negative experiences, they tend to use a self-immersing perspective, visualizing the events in the first person or through their own eyes. However, as people reflect on their feelings about an incident, they can also adopt a self-distancing perspective by viewing the experience from the perspective of an observer or from the vantage point of a “fly on the wall” (Libby and Eibach, 2002; Pronin and Ross, 2006; Vasquez and Buehler, 2007; Ayduk and Kross, 2008; Verduyn et al., 2012). A number of recent studies indicate that self-distancing can reduce the emotional arousal attached to events (Kross et al., 2005; Ayduk and Kross, 2008, 2010; Grossmann and Kross, 2010; Wisco and Nolen-Hoeksema, 2011; Verduyn et al., 2012). For example, reflecting on negative/positive experiences from a self- immersing perspective increases emotional arousal, whereas reflecting on these experiences from a self-distancing perspective attenuates it (Kross and Ayduk, 2017).

According to the notion of risk-as-feelings, emotional arousal plays a significant role in probability-weighting biases (Loewenstein et al., 2001; Rottenstreich and Hsee, 2001; Brandstätter et al., 2002; Pachur et al., 2014). For example, the bias of overweighting small probabilities can result from the anticipated emotional arousal after having won a very unlikely prize, while the bias of underweighting large-probability events can result from the anticipated emotional arousal after having failed to win a very likely prize (Brandstätter et al., 2002). More-intense emotional arousal has been shown to lead to larger biases of probability weighting (Faro and Rottenstreich, 2006; Kliger and Levy, 2008; Tyszka and Sawicki, 2011). Based on this, we would, therefore, expect self-distancing to reduce biases in probability weighting.

The effect of psychological distance on decisions made under risk has been investigated in other studies. Trautmann and van de Kuilen (2012), for example, explored the effect of psychological distance on valuations of buying and selling prices. They asked participants to either play lotteries or to trade them for a certain amount of money. The authors found that changing psychological distance had a weak effect on the valuation of outcome and probability lotteries. Their study, however, did not distinguish the probability weighting bias from the evaluation bias: the weak effect of psychological distance may result from the different effects of probability weighting bias or outcome value bias, or a combination of these two. We provide the first study that distinguishes between the two effects.

Our study is closely related to the literature on construal level theory, which claims that psychological distance from events leads to more abstract (vs. concrete) thinking (Trope et al., 2007; Trope and Liberman, 2010). Construal level has been shown to have a great influence on decisions under risk. Lermer et al. (2016) explored the effect of construal level on health-related risk estimation and found that, compared to concrete thinking, abstract thinking led to lower risk estimates for events. Some studies have shown that increasing psychological distance and assuming a more abstract construal level have similar effects on behaviors. For example, Lermer et al. (2015) found that abstract construal level resulted in greater risk-taking in the gain domain, while Sun et al. (2017) demonstrated that increased psychological distance makes people more risk-neutral (risk-seeking in the gain domain and risk-averse in the loss domain).

We want to emphasize that, although self-distancing is closely related to construal level, recent studies have shown distinct influences of self-distancing and construal level on emotion-based evaluation. Williams et al. (2014) demonstrated that increased psychological distance reduces both positive and negative effects and, therefore, improves the evaluation of negative experiences but hurts the evaluation of positive experiences. By contrast, with increased positivity, abstract thinking improves the evaluations of both positive and negative experiences. Thus, self-distancing and construal level can exhibit different effects on behaviors under risk.

The present study investigated whether self-distancing could reduce probability weighting biases. Participants were instructed to use either a self-distancing or a self-immersing strategy to regulate their reasoning when they completed a series of valuation tasks of probabilistic events. We hypothesized that participants using a self-distancing strategy would exhibit smaller biases of probability weighting compared to those using a self-immersing strategy and those in the baseline group.

## Methods

### Participants and design

As a part of a course requirement, 236 undergraduates (104 women; *M*_*age*_ = 21.13 years, *SD* = 1.70 years) who were enrolled in the Organizational Psychology class at East China Normal University participated in the experiment. They were randomly assigned to three groups to complete valuation tasks of probabilistic lotteries: the self-distancing group (*N* = 83; 40 women; *M*_*age*_ = 21.64 years, *SD* = 1.68 years); the self-immersing group (*N* = 84; 34 women; *M*_*age*_ = 21.30 years, *SD* = 1.63 years); and the baseline group (*N* = 69; 30 women; *M*_*age*_ = 20.32 years, *SD* = 1.52 years). The participants in the three groups did not differ in terms of gender and age, *p*s > 0.18. The Ethics Committee of East China Normal University approved the research procedures.

### Procedure and materials

Upon arriving at the laboratory, participants were informed that the experiment would investigate their decision-making habits. The participants were instructed to complete a valuation task of probabilistic lotteries, which were developed by Borcherding et al. (1991). In the task, participants were shown 28 binary prospects (i.e., a lottery with probability distributions over two outcomes) that were used by Tversky and Kahneman (1992) (these can be found in Table 1). Each prospect consisted of a probability that *p*_*i*_ would obtain RMB *x*_i_ and a probability that (1–*p*_i_) would obtain RMB *y*_*i*_^1^. In different prospects, *p*_*i*_ took the values of 1, 5, 10, 25, 5, 75, 90, 95, or 99%, while (*x*_i_, *y*_*i*_) took the values of (50, 0), (100, 0), (200, 0), (400, 0), (100, 50), (150, 50), or (200, 100). Note that *x*_i_ was always significantly greater than *y*_*i*_. Participants were asked to indicate the amount of cash they would need in order to be indifferent between definitely receiving that cash amount and taking the lottery^2^. Both the order of probabilities and the outcomes were counterbalanced across the participants. We did not impose time constraints on completion of the valuation tasks.

**Table 1:** Median certainty equivalents for different groups.

***x*_*i*_**	***y_*i*_***	**Treatment**	***p_*i*_***								
			**0.01**	**0.05**	**0.10**	**0.25**	**0.50**	**0.75**	**0.90**	**0.95**	**0.99**
50	0	EV	–	–	5	–	25	–	45	–	–
		Distancing			7		24		42		
		Baseline			9		24		41		
		Immersing			9		23		40		
100	0	EV	–	5	–	25	50	75	–	95	–
		Distancing		10		24	45	63		84	
		Baseline		10		29	46	65		88	
		Immersing		13		26	39	59		80	
200	0	EV	2	–	20	–	100	–	180	–	198
		Distancing	6		19		87		147		195
		Baseline	5		25		83		155		192
		Immersing	8		24		83		138		191
400	0	EV	4	–	–	–	–	–	–	–	396
		Distancing	6								389
		Baseline	9								374
		Immersing	9								377
100	50	EV	–	–	55	–	75	–	95	–	–
		Distancing			57		74		87		
		Baseline			56		77		88		
		Immersing			60		74		84		
150	50	EV	–	55	–	75	100	125	–	145	–
		Distancing		61		72	90	111		134	
		Baseline		61		80	95	115		136	
		Immersing		64		74	88	107		130	
200	100	EV	–	105	–	125	150	175	–	195	–
		Distancing		113		126	143	169		184	
		Baseline		109		127	145	163		184	
		Immersing		116		129	142	162		180	

*EV represents the expected value of the lottery. According to our hypothesis, the value under the “distancing” group should be closer to EV compared to the “immersing” and baseline groups*.

Based on the method used in previous studies, participants were instructed to use different cognitive strategies while making decisions (Ayduk and Kross, 2008; Kross and Grossmann, 2012; Kross and Ayduk, 2017). In the self-distancing group, participants were instructed to “*consider each gamble in a rather distanced way; take a certain distance from what happens; look at what happens [in]each gamble from the perspective of an external observer*.” In the self-immersing group, participants were instructed to “*consider each gamble with an emotional interest in it; enter into what happens; look at what happens [in] each gamble from the perspective of an involved participant*.” In the neutral group, participants were instructed simply to finish the task without asking them to use any strategy.

After finishing the task, the participants rated the perspective that they had adopted during the evaluation phase (1 = *immersed entirely*; 9 = *distanced entirely*). Thereafter, demographic information (i.e., gender and age) was collected. Finally, the participants were thanked, debriefed, and paid. The experiment lasted for approximately an hour.

## Results

### Manipulation checks

The differences between any two groups in the self-rating task were statistically significant—*F*_(2, 233)_ = 166.90. Participants in the self-distancing group reported more distancing (*M* = 7.27, *SD* = 1.47) than those in the baseline group (*M* = 4.01, *SD* = 1.88) and those in the self-immersing group (*M* = 2.92, *SD* = 1.44) in the self-rating task, *p*s < 0.001. Participants in the baseline group reported more distancing than those in the self-immersing group, *p*s < 0.001, suggesting the successful manipulation of self-distancing.

### Certainty equivalents

Table 1 reports the median certainty equivalents indicated by participants in the different groups in each scenario, along with the expected cash values of the lotteries. Consistent with Tversky and Kahneman (1992), participants in both groups reported certainty equivalents greater than the expected cash value for *p* < 0.5, while the reported certainty equivalents were less than the expected cash value when *p* ≥ 0.5^3^^,^^4^.

Note that reporting a certainty equivalent equal to the expected cash value maximizes the expected payoff. To understand this, consider a lottery that gives 100 with *p* = 0.5 and nothing otherwise. A certainty equivalent of 40 would mean that, when faced with the choice between the lottery and a guaranteed 45, the person would choose the 45 for sure, which falls short of the lottery's EV. Analogously, with a certainty equivalent of 60 and a choice between the lottery and a certain amount of 55, the person would choose the lottery, which gives an EV < 55. If self-distancing improves decision making by reducing biases, the reported value under the “self-distancing” condition should be closer than both the “self-immersing” and baseline conditions to the EV. Indeed, in almost all cases, the participants in the distancing group reported certainty equivalents closer to the payoff-maximizing values, indicating that self-distancing leads to decisions that maximize expected payoffs. However, the improvement in the decisions can be due to either the reduction in the probability weighting bias or the valuation bias. In the next subsection, we disentangle these two effects and demonstrate that the effect of self-distancing is mainly the reduction of the probability weighting bias.

### Probability weighting bias

Based Tversky and Kahneman (1992) estimation method, we modeled our data to estimate participants' probability weighting function. Specifically, the cash equivalent (CE) indicated by the participant was determined as follows:

(1) $$v(CE)=w(p_{i})v(x_{i})+[1-w(p_{i})]v(y_{i})$$

This function reflects that the cash equivalent was determined by participants' subjective weight of probability *p*_*i*_ [i.e., *w*(*p*_*i*_)] and subjective value of outcomes *x*_*i*_ [i.e., *v*(*x*_*i*_)]. *w*(*p*_*i*_), *v*(*x*_*i*_), and *v*(*y*_*i*_) are defined, respectively, as follows:

(2) $$w(p)=\frac{p^{\gamma }}{{\left(p^{\gamma }+{\left(1-p\right)}^{\gamma }\right)}^{1/\gamma }}$$

(3) $$v(x)=x^{a}$$

(4) $$v(y)=y^{a}$$

In Equation (2), the parameter γ is the measure of the curvature of the probability weighting function (γ > 0). When γ = 1, participants' subjective probability is the same as their objective probabilities (i.e., no bias of probability evaluations). When 0 < γ < 1, the participants overweight small probabilities and underweight large probabilities. A lower γ indicates a more pronounced bias in probability weighting.

In Equations (3) and (4), the parameter α reflects a participant's diminishing sensitivity to the change in the outcome (α > 0). When 0 < α < 1, the participant becomes less sensitive to the change in the outcome as the size of the change increases. A smaller α indicates a more pronounced diminishing sensitivity.

**Figure 1:**
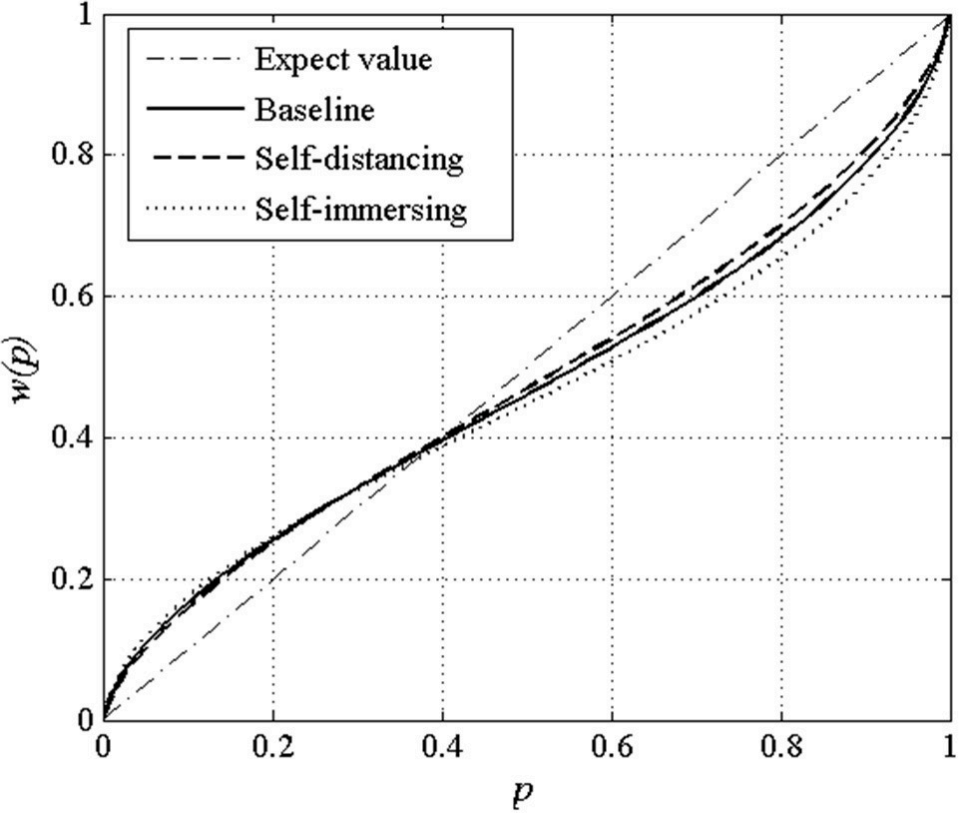
Probability weighting function *w(p)* for the baseline group, self-distancing group, and self-immersing group.

We estimated the parameters in Equations (2–4) for each participant using a non-linear least-squares regression procedure. To have an idea of the model's goodness of the fit, the average Root Mean-Squared Error (RMSE) under the baseline group, the self-distancing group, and the self-immersing group are 14.40, 22.57, and 22.03, respectively, and the average R-squared in these three groups are 0.96, 0.92, and 0.91, respectively. The high R-squared values indicate that the model explains the data well. The median values of the estimated γ in the baseline group, the self-distancing group, and the self-immersing group are 0.70, 0.75, and 0.67, respectively. Using the non-parametric Mann–Whitney tests, we find that the differences between any two groups are statistically significant (baseline vs. self-distancing, *p* = 0.004; baseline vs. self-immersing, *p* = 0.009; self-distancing vs. self-immersing, *p* < 0. 0.001). The estimated values are consistent with our hypothesis: compared to the participants in the baseline group, those in the self-distancing group exhibit lower probability weighting bias, while participants in the self-immersing group exhibit higher bias. To help visualize the probability weighting bias in the three groups, Figure 1 illustrates the probability weighting function that is constructed by the medians of γ.

The median values of α under the baseline group, the self-distancing group, and the self-immersing group are 0.93, 0.86, and 0.86, respectively. Interestingly, we find evidence that participants in the self-distancing group exhibit higher curvature (α) in the valuation function than those in the baseline group (*p* = 0.006, Mann–Whitney test). Note that both curvature in the value function (α < 1) and non-linearity in the probability weighting function (γ < 1) lead to decisions that do not maximize the expected cash payoff.

## General discussion

The present study aims to examine whether self-distancing could reduce the probability weighting biases. Consistent with previous studies (e.g., Tversky and Kahneman, 1992), we found that participants exhibited biases of overweighting small probabilities and underweighting large probabilities. Importantly, we found that when participants used a self-distancing strategy to regulate their reasoning, their probability weighting functions exhibited less curvature. These findings suggest that self-distancing can reduce the probability weighting biases.

Comparing our study with Lermer et al. (2016) yields some insights. While the two studies have documented two mechanisms that can reduce probability weighting biases, it appears that self-distancing and a changing construal level have distinct effects. We showed that self-distancing reduced both biases of overweighting small probabilities and underweighting large probabilities, whereas Lermer et al. (2016) revealed that abstract thinking reduced the estimation of both small and large probabilities. We speculate that *emotion arousal and valence* play important roles in the distinct effects. Williams et al. (2014) found that self-distancing improves evaluations of negative experiences by reducing the arousal of a negative effect, but it hurts evaluations of positive experiences by reducing the arousal of a positive effect. In contrast, abstract thinking increases positivity, improving evaluations for both positive and negative experiences alike. The notion of risk-as-feelings (Loewenstein et al., 2001) probability weighting biases implies that more positive emotional arousal after having won a very unlikely prize can lead to a larger bias of overweighting small probabilities, while more negative emotional arousal after having failed to win a very likely prize can lead to a larger bias of underweighting large probabilities (Brandstätter et al., 2002). This explains why self-distancing and construal level have exhibited different effects on probability weighting biases. We call for future studies to further investigate the difference between these two mechanisms.

Our estimation based on Tversky and Kahneman (1992) allows us to disentangle the biases in valuation and in probability weighting. Note that α and γ have different influences on behaviors. Valuation biases (α < 1) always lead to risk aversion and understatement of certainty equivalents, while probability weighting biases (γ < 1) lead to overstatement of certainty equivalents when the probability of gains is small but to understatement when the probability is big. Although our findings showed that self-distancing reduced the probability of weighting biases, it increased outcome value bias (e.g., the parameter α in the self-distancing group was lower than that in the baseline group). This provides a reasonable explanation for the weak effect of psychological distance on price valuations in the study of Trautmann and van de Kuilen (2012): increasing psychological distance from the self may reduce the probability weighting biases but may increase outcome value bias, making the total effect of psychological distance on valuations weaker.

While the current study has provided strong evidence that self-distancing can reduce the biases in probability weighting, we acknowledge that it has limitations. The analysis of our data rests on the assumption that participants make decisions using the cognitive processes postulated by prospect theory (i.e., weighting and summing). This assumption has been challenged by the heuristic assumption (Brandstätter et al., 2006). According to the heuristic assumption, it is possible that participants rely on some simple heuristics without weighting the probabilities of different events. That is, the self-distancing group and the self-immersing group may have used different heuristics, making the participants act “as if” they exhibited different degrees of probability weighting biases in their decision making. In the current study, we used a valuation task to measure probability weighting biases. Although both valuation tasks and decision tasks were often used to study probability weighting bias (Rottenstreich and Hsee, 2001; Gneezy et al., 2006), studies have suggested differences between these two methodologies (Harbaugh et al., 2010). We call for future studies to investigate whether the effect of self-distancing on probability weighting bias is robust using a decision task.

To summarize, our results offer evidence for the power of self-distancing to reduce biases in probability weighting, which has important practical implications. We believe that, by using a self-distancing strategy, investors, doctors, and budgeters can put more-rational weight on probability in investments, medical diagnoses, and budgets.

## Ethics statement

This study was carried out in accordance with the recommendations of Ethics Committee of East China Normal University with written informed consent from all subjects. All subjects gave written informed consent in accordance with the Declaration of Helsinki. The protocol was approved by the Ethics Committee of East China Normal University.

## Author contributions

QS, LS, and FH: generating the ideas and design. HZ and QS: revising the article; HZ: refining the design and analysis of data. Additionally, we also thank Qian Zheng and Jianhua Jiang from Zhijiang College of Zhejiang University of Technology in collecting data.

### Conflict of interest statement

The authors declare that the research was conducted in the absence of any commercial or financial relationships that could be construed as a potential conflict of interest.

## Footnotes

^1^RMB is the currency used in China, with the exchange rate of 1RMB = US$0.15 at the time of the experiment.

^2^In the current study, we directly asked the participants to state their certainty equivalents using valuation tasks, whereas Tversky and Kahneman (1992) presented the participants a series of binary choice tasks and inferred the certainty equivalents from these tasks. Although both types of tasks have been used to measure the probability weighting bias (Rottenstreich and Hsee, 2001; Gneezy et al., 2006), studies have suggested that these two methodologies may lead to behavioral differences (Harbaugh et al., 2010). Further information on this is provided in the discussion of the experimental results.

^3^There are two exceptions: when *p* = 0.25, (x, y) = (100, 0) and *p* = 0.25, (x, y) = (150, 50), the reported certainty equivalents are less than the expected cash value. However, the deviations are small (< 4% of the expected cash value) and do not influence the results. Tversky and Kahneman (1992) reported similar values for these two scenarios.

^4^We also provide the mean certainty equivalents with standard errors in the Table A1 in Appendix. The patterns we observe for the means are similar to the patterns for the medians.

## Appendix

**Table A1:** Mean cash equivalents (standard errors) for all scenarios.

***x*_i_**	***y_i_***	**Treatment**	***p_i_***								
			**0.01**	**0.05**	**0.10**	**0.25**	**0.50**	**0.75**	**0.90**	**0.95**	**0.99**
50	0	EV	–	–	5	–	25		45	–	–
		Distancing			7.64 (0.28)		23.22 (0.37)		41.66 (0.35)		
		Baseline			8.77 (0.29)		23.36 (0.52)		40.04 (0.66)		
		Immersing			9.11 (0.37)		22.88 (0.39)		39.31 (0.41)		
100	0	EV	–	5	–	25	50	75	–	95	–
		Distancing		10.55 (0.46)		23.82 (0.66)	43.34 (0.74)	61.10 (0.76)		82.90 (0.59)	
		Baseline		10.41 (0.34)		27.39 (0.69)	43.62 (0.97)	62.28 (1.21)		85.28 (1.12)	
		Immersing		13.30 (0.60)		25.44 (0.84)	37.43 (0.77)	57.81 (0.81)		79.26 (0.70)	
200	0	EV	2	–	20	–	100	–	180	–	198
		Distancing	6.65 (0.36)		18.69 (1.05)		83.30 (1.43)		144.23 (1.34)		192.72 (0.69)
		Baseline	5.46 (0.35)		25.49 (0.86)		78.86 (1.87)		149.49 (2.41)		188.23 (1.38)
		Immersing	9.52 (0.69)		24.43 (1.46)		79.06 (1.57)		136.39 (1.55)		188.73 (0.90)
400	0	EV	4	–	–	–	–	–	–	–	396
		Distancing	7.22 (0.72)								385.30 (1.68)
		Baseline	9.67 (0.61)								368.13 (2.63)
		Immersing	13.21 (1.40)								374.96 (1.58)
100	50	EV	–	–	55	–	75	–	95	–	–
		Distancing			56.83 (0.61)		74.04 (0.32)		85.76 (0.78)		
		Baseline			56.35 (0.20)		76.45 (0.41)		86.45 (0.48)		
		Immersing			61.31 (0.66)		73.26 (0.37)		82.86 (0.76)		
150	50	EV	–	55	–	75	100	125	–	145	–
		Distancing		61.46 (0.38)		72.42 (0.47)	90.40 (0.44)	111.08 (0.60)		133.64 (0.51)	
		Baseline		61.25 (0.30)		79.30 (0.34)	93.22 (0.61)	113.46 (0.91)		134.71 (0.87)	
		Immersing		64.65 (0.52)		73.73 (0.46)	88.58 (0.25)	106.94 (0.69)		129.43 (0.58)	
200	100	EV	–	105	–	125	150	175	–	195	–
		Distancing		112.81 (0.56)		126.63 (0.42)	143.98 (0.52)	169.43 (0.54)		183.04 (0.45)	
		Baseline		109.12 (0.40)		126.83 (0.37)	143.75 (0.57)	161.16 (0.91)		182.29 (0.85)	
		immersing		116.40 (0.54)		129.57 (0.46)	141.86 (0.32)	162.12 (0.53)		178.81 (0.79)	
